# The Spillover Effect of Life Satisfaction on Customer Satisfaction: The Mediating Role of Service-Oriented Organizational Citizenship and the Moderating Role of Competition Climate

**DOI:** 10.3390/bs12080276

**Published:** 2022-08-10

**Authors:** Yunyun Luan, Meizhen Lv, Lei Wang

**Affiliations:** School of Psychological and Cognitive Sciences and Beijing Key Lab for Behavior and Mental Health, Peking University, Beijing 100871, China

**Keywords:** employee life satisfaction, spillover, customer service satisfaction, group competition climate, organizational citizenship behavior

## Abstract

In the field of organizational management, research on employees’ life experiences is insufficient. It remains unclear how employees’ non-work experiences affect customer service quality. Building on the spillover theory (an individual’s experience in one domain can be transferred to another domain) and the conservation of resources theory (individuals are motivated to protect their current resources and to acquire new resources), we aim to examine the impact of service employee’s life satisfaction on customer satisfaction while considering service-oriented organizational citizenship behavior (the discretionary extra-role behavior demonstrated by employees) as a mediating variable. To test our hypothesis, we collected multi-wave, multilevel, multisource data of 209 customer service employees from an insurance company. The results showed that service-oriented organizational citizenship behavior was an important mechanism by which employees’ overall life satisfaction positively affected customer satisfaction. In addition, this mediating effect was weaker for employees in higher competition climate groups. The study illustrates the critical contribution of the overall well-being of personnel to organizational business results.

## 1. Introduction

An increasing number of studies have revealed that employees’ work experience is of great significance to business results [1,2,3]. However, in the field of organizational management, research on employees’ life experiences has been insufficient, and few enterprises fully appreciate how employees’ life satisfaction affects their behavior and performance in the workplace [4]. Extant studies on the spillover effect of non-work domains on the work domain still have the following limitations: (1) previous studies have focused on the spillover of family experience into the workplace [5,6,7] but have ignored the impact of other meaningful life experiences on work [8]. (2) Extant studies have used life satisfaction as an outcome variable to investigate its antecedents [9,10], but the research on life satisfaction itself as the predictive variable has been inadequate [4]. (3) Though the vast majority of studies have explored the relationship between life satisfaction and job satisfaction [11,12], it remains unclear how employees’ life experience affects their job performance in such areas as customer satisfaction [4]. Given these deficiencies, this paper will pay attention to the spillover effect of employees’ overall life experience in the workplace and focus on exploring the relationship between employees’ overall life satisfaction and customer satisfaction.

Based on the model of work–family enrichment [13] and the conservation of resources theory (the COR Theory) [14,15], this paper proposes a mediating model which states that life satisfaction improves customer satisfaction by promoting service-oriented organizational citizenship behavior (service-oriented OCB) [16].

In accordance with the work–family enrichment model, we posit that higher life satisfaction leads to more organizational citizenship behavior [17] through increasing employee job satisfaction [16,18,19]. Moreover, according to the COR theory, individuals possessing rich job resources are more likely to be highly engaged in work [20,21,22,23] and thus demonstrate more organizational citizenship behavior [24,25]. In addition, service-oriented OCB is vital for customer service quality [26,27,28]. Therefore, it is theoretically and practically important to investigate whether and how service-oriented OCB affects the relationship between life satisfaction and customer satisfaction.

Furthermore, the effect of employees’ life satisfaction upon customer satisfaction may also be affected by boundary conditions. Based on the COR theory, we propose that group competition climate is a critical boundary condition in this regard. A high group competition climate increases the reduction in support from coworkers [29] and the loss of control over success [30], both of which are essential work resources according to the COR theory [15]. According to previous research, individuals in a competitive group are more likely to be unable to obtain sufficient resources following an investment of work resources [31,32,33]. Therefore, this study proposes that an increase in group competition climate makes employees more vulnerable to a loss of work resources, which weakens the positive effects of life satisfaction on customer satisfaction.

This study makes many theoretical contributions. First, we expand the understanding of the critical role of employees’ non-work experience on customer experience. We complement existing research on how employees’ life satisfaction relates to customer satisfaction [4,34]. Second, our study enriches the COR theory by clarifying the positive effect of non-work experience on customer service quality through the mediating role of service-oriented OCB. In particular, it broadens our understanding of the COR theory as a theoretical framework to explain how personal resources affect service-oriented OCB. Third, we identified a negative moderating effect of group competition climate on service performance, which contradicts the historical view of its positive role [35].

## 2. Theoretical Background

Spillover refers to the connection mechanism between work and life [36]. Spillover theory proposes that an individual’s experience in one domain can be transferred to another [37]. Some researchers have proposed a division of spillovers into two categories: (1) horizontal spillover, which refers to the influence of affect in one life domain on a neighboring domain; and (2) vertical spillover, refers to the spillover effect between the most superordinate domain and the subordinate domains [38].

In accordance with spillover theory, this study proposes that investigating the impact of life on work from the perspective of vertical spillover is a topic worthy of more in-depth discussion, compared with horizontal spillover. To date, there have been numerous studies on horizontal spillovers, mainly focusing on discussing “family-to-work” spillovers [39,40,41]. However, employees also encounter a large number of other meaningful life events outside the family domain, relating to such matters as health conditions [42], financial security [8], social relations, and leisure satisfaction [43], which also affect the workplace experience of the individual in complex and as-yet-unexplored ways [44]. To fully understand the experience of employees in the workplace, it is necessary to recognize their overall experience outside the workplace [44,45]. Therefore, this study proposes to use general life satisfaction [46], which is an indicator of global quality of life [47] to explore the spillover effect of life on work from a broader perspective.

### 2.1. Life Satisfaction and Customer Satisfaction

In accordance with spillover theory and in line with existing research, we propose that life satisfaction will correlate with customer satisfaction through job satisfaction and job burnout, respectively. On the one hand, life satisfaction is positively related to job satisfaction [12,48], and job satisfaction is positively associated with customer satisfaction [18,49,50,51]. On the other hand, life satisfaction is negatively related to job burnout [52,53], and job burnout is negatively associated with customer satisfaction [54,55].

First, the relationship between life satisfaction and job satisfaction can be supported from theoretical and empirical perspectives. Theoretically, the work–family spillover theory [13] posits a relationship between life satisfaction and job satisfaction. This theory holds that an individual’s experience in one role in life will promote a similar experience in another. Specifically, individuals with happy lives are much more likely to be satisfied with their jobs, and vice versa [4,11,56]. Previous studies have supported a correlation and causal relationship between life satisfaction and job satisfaction. A meta-analysis by Bowling et al. [12] found that the correlation coefficient between life satisfaction and job satisfaction ranged from 0.16 to 0.68 [12] As for the direction of causality in the relationship, previous research suggests that the relationship is reciprocal, though there is a stronger influence of life satisfaction on subsequent job satisfaction than of job satisfaction on subsequent life satisfaction [11,12]. Therefore, we posit that employees with high overall life satisfaction will also have high job satisfaction.

Furthermore, the relationship between job satisfaction and customer satisfaction has also been empirically supported. It has been found that job satisfaction is positively associated with customer-oriented behavior [49,57]. The results of a meta-analysis also support a significant positive correlation between employee job satisfaction and customer satisfaction across various industries [58]. At the same time, one study found that job satisfaction can significantly predict customer satisfaction [50].

Second, life satisfaction can alleviate job burnout by reducing negative emotions. Specifically, life satisfaction is a cognitive component of subjective well-being (SWB) [46] and the other two affective components are positive affect and negative affect [47]. Life satisfaction is positively related to positive affect [59,60] In other words, a higher level of life satisfaction can enhance positive affect and alleviate job burnout by reducing emotional exhaustion [61]. Thus, we propose that life satisfaction is related to reduced job burnout [53].

At the same time, job burnout has a negative impact on customer satisfaction. For example, job burnout has a negative impact upon employees’ work attitude [62]; reduces employees’ commitment to customers [63]; diminishes the display of genuine emotions by employees towards customers [64]; negatively affects customer-oriented behavior [65]; and damages the creativity of frontline service personnel [66]. In conclusion, the higher the degree of employee burnout, the more likely it will produce a negative impact on customer satisfaction [54,55].

Taken together, we propose that life satisfaction can promote customer satisfaction by increasing job satisfaction and reducing job burnout. Accordingly, we hypothesize:

**Hypothesis** **1** **(H1).** *Life satisfaction is positively related to customer satisfaction*.

### 2.2. The Mediating Role of Service-Oriented Organizational Citizenship Behavior

Organizational citizenship behavior (OCB) refers to extra-role behavior demonstrated by an individual at the workplace, which is discretionary, not directly or explicitly recognized by the formal reward system, and aggregates to promote the effective functioning of the organization [67]. Service-oriented OCB refers to the discretionary behavior performed by customer-contact employees in a service organization that surpasses their formal role prescriptions [68]. In contrast to the prior concept of organizational citizenship behavior [67], which is widely applicable across different types of organizations and positions, service-oriented OCB is mainly concerned with service personnel in contact with customers [18].

Based on the work–family enrichment model and the conservation of resources theory, we propose that service-oriented OCB may explain the internal mechanism of the relationship between life satisfaction and customer satisfaction. In other words, employees with higher life satisfaction are more willing to provide customers with services beyond their responsibilities, resulting in higher customer satisfaction.

First, our view is based on the work–family enrichment theory [13]. The theory holds that a mutual positive spillover between work and family can be achieved through an instrumental path and an affective path [69]. The instrumental path operates through resources, so that “a resource can be transferred directly from Role A to Role B, thereby enhancing performance in Role B” [13] (p. 80). The emotional path operates through positive emotion, so that “a resource generated in Role A can promote positive affect within Role A, which, in turn, produces performance and positive affect in Role B” [13] (p. 80). Therefore, we may expect that individuals with high quality of life will spill over resources and positive emotions from life to work, which will promote their performance and positive emotions at work [70] and, in turn, improve the job satisfaction of employees [71]. Moreover, employee job satisfaction can be used as an important attitude factor to predict service-oriented OCB [16,18,19] because proactive behavior at work is often driven by a positive affect brought out by job satisfaction [72,73].

Second, our view is also based on the COR theory. The theory posits that individuals are motivated both to protect their current resources (conservation) and to acquire new resources (acquisition) [14]. The definition of “resources” refers to anything perceived by the individual to help attain his or her goals [22]. The theory also holds that those who possess resources are more capable of gaining more, and, in addition, that initial resource gain begets future such gain, thus generating “gain spirals” [15], meaning that individuals with high life satisfaction are more likely to accumulate resources at work. According to previous studies, individuals with rich working resources tend to have higher work engagement [20,21,22,23], and highly engaged employees are more willing to do work beyond their duties [24,25]. In other words, life satisfaction can positively predict organizational citizenship behavior. Therefore, we propose that service personnel who are more satisfied with life may exhibit more willingness to go beyond the call of duty to provide customers with extra services.

In conclusion, life satisfaction can predict service-oriented OCB in two ways: (1) life satisfaction positively affects job satisfaction, which, in turn, promotes service-oriented OCB; (2) life satisfaction positively impacts work engagement by increasing work resources, and work engagement positively impacts service employees’ OCB. Based on the above arguments, we formulate the following hypothesis:

**Hypothesis** **2** **(H2).** *Employees’ life satisfaction will positively affect their service-oriented OCB*.

Furthermore, organizational citizenship behavior is vital for promoting customer service quality [26]. For example, organizational citizenship behavior can improve customer satisfaction [27,28] and customer loyalty [72]. The main reason is that no one can specify the full range of duties a service employee might have to perform in response to unpredictable customer requests [74,75]. Therefore, the delivery and service carried out by service personnel beyond specified job standards or the expectations of customers will significantly affect customers’ perception of service quality [76]. We believe that service-oriented OCB will serve a similar function.

To sum up, we further formulate the indirect effect hypothesis of life satisfaction and customer satisfaction:

**Hypothesis** **3** **(H3).** *Service-oriented OCB plays a mediating role in the relationship between life satisfaction and customer satisfaction*.

### 2.3. The Moderating Role of Group Competition Climate

Although we predict that life satisfaction will positively impact customer satisfaction, the degree of this impact may vary with differing levels of group competition climate in the workplace. Drawing upon the COR theory, we propose that, compared with a highly competitive team climate, a relaxed and cooperative team climate is more likely to enable a life-satisfied employee to provide high-quality customer service.

The COR theory explains the negative impact of a competitive group climate on an individual’s work resources. First, a high group competition climate hinders employees from obtaining two necessary work resources: (1) peer support; and (2) a sense of control over work results. Group competition climate refers to the extent to which employees perceive organizational rewards to be contingent on comparing their performance against that of their peers [77]. Compared with a low competition climate, a high competition climate means that (1) relationships among team members are more conflictual [78] and mutually exclusive, rather than mutually supportive and cooperative [29]; (2) uncertainty of achieving performance increases [30], indicating to employees that future success does not entirely depend on themselves, which makes them feel less of a sense of control over work results. The COR theory states that support from coworkers and a feeling of control over success are both vital work resources [15]. Thus, the availability of these work resources will decline with an increased competition climate.

Second, a high group competition climate reduces the ability of individuals to gain sufficient resources. Competition implies that there might be a lack of resource gains, such as promotion opportunities or job security, following significant resource investment of time and energy [31,32,33]; that is, resource investment is more salient than resource gains. This chronic resource loss is a principal explanatory mechanism for understanding the process of work stress and burnout [15]. A meta-analysis review has shown empirical evidence for the negative impact caused by a competitive group climate through the continuous depletion of individual resources. It was found that individuals experience increased levels of stress regardless of their own level of trait competitiveness when individual perceptions of a competitive climate increased [30].

Based on the above arguments, we construct the following hypothesis:

**Hypothesis** **4** **(H4).** *Group competition climate moderates the within-individual indirect relationship between life satisfaction and customer satisfaction mediated by service-oriented OCB, such that the relationship between service-oriented OCB and customer satisfaction will be weaker for groups with a higher compared to those with a lower competition climate*.

**Figure 1:**
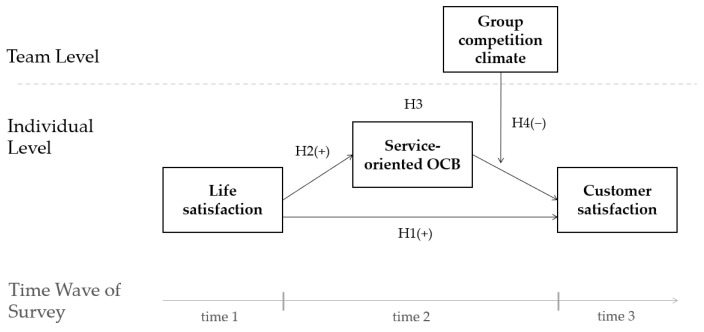
A Conceptual Model of the Hypotheses.

The hypothesized model can now be illustrated, as shown in Figure 1.

## 3. Materials and Methods

### 3.1. Sample and Procedure

Participants in the current study were recruited from the call center of a large Chinese insurance company. They deliver service to customers and are required to attain specified performance objectives, including an indicator of customer satisfaction. Service employees in the call center work as a team so that they are responsible for achieving team goals and individual goals every month. The research team and the human resource management department of the company screened all full-time employees and sent an invitation letter to these 385 employees. Two hundred thirty-seven employees voluntarily participated in the survey, and 28 were eliminated from the analyses due to missing archival data on performance, leaving a final sample of 209 employees (response rate = 54.3%). These employees nested in 29 groups (response rate = 64.1%) with an average group size of 7.21, meeting the requirement that the smallest acceptable number for groups should be 20, with a minimum of five individuals per group for observation purposes [79]. The majority of the sample was female (85.6%), the average age of participants was 26.21 years (*SD* = 3.16), and the average job tenure was 21.80 months (*SD* = 23.86).

We used a time-lagged research design to reduce the potential for common-source and common-method biases and collected data at three time lags. At Time 1 (T1, beginning of the month t), life satisfaction and demographic variables were obtained from employees. One month later, at Time 2 (T2, beginning of the month t + 1), an assessment of competitive psychological climate and service-oriented OCB were obtained from employees who completed the survey at Time 1. We then collected employees’ archival data of service quality ratings at Time 3 (T3, end of the month t + 1).

### 3.2. Measures

***Life Satisfaction***. Life satisfaction was measured by a five-item scale developed by Diener (1985) [46]. Sample items include “The conditions of my life are excellent.” and “I am satisfied with my life.”. The possible responses range from 1 (strongly disagree) to 7 (strongly agree). The higher the score, the more satisfied the employee feels with their life. The coefficient α for life satisfaction was 0.86.

***Group Competition Climate.*** Group competition climate was measured using a four-item scale developed by Brown (1998) [77]. Sample items include “My manager frequently compares my results with those of other peers.” and “My co-workers frequently compare their results with mine.”. Possible responses range from 1 (strongly disagree) to 7 (strongly agree). Higher scores represent higher competition levels, as perceived by the employee. The coefficient α for group competition climate was 0.86. To aggregate the individual perceptions of competition climate to a workgroup level, we assessed the average within-group agreement (rwg.j) and the intra-class correlations for this scale. The result showed that the average rwg (j) across groups was 0.51 and that ICC (1), the percentage of employee perceived competition level variance due to between-group factors, was 0.14.

***Service-oriented OCB.*** Service-oriented OCB were measured by a 16-item scale proposed by Bettencourt (2001) [18]. This scale includes three service-oriented OCB dimensions: loyalty (five items), participation (five items) and service delivery (six items). The possible responses range from 1 (strongly disagree) to 7 (strongly agree). The higher the score, the more service-oriented OCB demonstrated by the employee. The coefficient α for service-oriented OCB was 0.96.

***Customer Satisfaction.*** The measurement of customer satisfaction used the real-time feedback rating of the customer after the delivery of service by the employee. The human resource department then converted this rating into a percentage score used in this study. A higher score indicates a more satisfying customer experience.

***Control Variables.*** We controlled for employee demographic information, such as gender (male or female), age (in years) and job tenure (in months), since age has been found to be associated with OCB [80], and gender and job tenure have been found to be associated with customer satisfaction [81,82]. The data were collected through the survey questionnaire and were double-checked with the archival data provided by HR.

### 3.3. Analytical Procedure

We expected that, at the individual level, the life satisfaction of employees would positively predict customer satisfaction, and that the effect could be mediated by the service-oriented OCB. We used linear regression to test the direct effect with SPSS and used Preacher and Hayes’ (2008) bootstrapping procedure [83] (5000 iterations) and corresponding SPSS macro to test for the mediation effect.

We then tested the significance of paths of the multilevel mediated moderation model by considering the cross-level moderating role of group competition climate. Mplus was used to build the hierarchical linear model, which helps separate the effects of between- and within-group variance on the dependent variable [84] and construct bias-corrected confidence intervals through a bootstrapping approach. The parameters were estimated using maximum-likelihood estimation. The individual-level predictors were group-mean centered, and the group-level predictor was grand mean centered [85].

## 4. Results

### 4.1. Descriptive Statistics and Correlations

Means, standard deviations, scale reliabilities, and correlations were calculated (see Table 1). It can be seen that the correlation between life satisfaction and service-oriented OCB was significant and positive (*r* = 0.15, *p* < 0.05). The correlation of service-oriented OCB and customer satisfaction was also positive, though only marginally significant (*r* = 0.13, *p* < 0.10). Table 1 also shows that group competition climate was positively associated with service-oriented OCB (*r* = 0.51, *p* < 0.01). Furthermore, there was a marginally significant interaction between age and customer satisfaction (*r* = 0.12, *p* < 0.10) and a significant and positive correlation between tenure and customer satisfaction (*r* = 0.19, *p* < 0.01).

### 4.2. Testing the Construct Validity

Before hypothesis testing, we conducted a set of confirmatory factor analyses (CFAs, see Table 2) to establish convergent and discriminant validity for the multi-item constructs used in this study. Since customer satisfaction was independently measured by customers, it was not included in the CFA model. In the first CFA, we examined a three-factor model, with those factors being life satisfaction, service-oriented OCB and group competition climate. The hypothesized model showed a good fit to the data (*χ*^2^ (232) = 411.55, RMSEA = 0.06, SRMR = 0.06, CFI = 0.96, TLI = 0.95).

We compared the three-factor model with several alternative models to test the discriminant validity. In the two-factor model, service-oriented OCB and group competition climate were combined into a single factor. In the single-factor model, all variables were combined into one factor. As shown in Table 2, the hypothesized three-factor model fitted the data considerably better than any alternative models.

### 4.3. Hypothesis Testing

Hypothesis 1 posited the effects of life satisfaction on customer satisfaction, which was tested by linear regression with SPSS. Model 1 (Table 3) showed that after controlling for gender, age and job tenure, the relationship between life satisfaction and customer satisfaction was insignificant (*B* = −0.04, *t* = −0.22, *p* > 0.05). Thus, hypothesis 1 was not supported.

In support of Hypotheses 2 and 3, we used sample bootstrapping tests of indirect effects concerning the mediating role of service-oriented OCB. Model 2 (Table 3) showed that, after controlling for gender, age and tenure, life satisfaction was significantly related to service-oriented OCB (*B* = 0.12, *t* = 2.22, *p* < 0.05), The 95% bias-corrected bootstrapped confidence interval does not include zero (CI = [0.013, 0.232]), indicating that the effect of life satisfaction on service-oriented OCB was significantly positive. Thus, Hypothesis 2 was supported.

After controlling for gender, age and tenure, the direct effect of life satisfaction on customer satisfaction was nonsignificant (*B* = −0.09, *t* = −0.55, *p* > 0.05, CI = [−0.40, 0.23]). However, the indirect effect of life satisfaction on customer satisfaction through service-oriented OCB was significantly positive (*B* = 0.05, *SE* = 0.04, CI = [0.003, 0.150]). Thus, service-oriented OCB completely mediated the relationship between life satisfaction and customer satisfaction. Hypothesis 3 was therefore supported.

Table 3 presents the analysis results for testing Hypotheses 1 to 3.

Extending Hypothesis 3, we suggested that the indirect relationship between life satisfaction and customer satisfaction would be weaker for high-level competition climate groups. To test the moderated mediation effects, we proceeded to multilevel analysis in Mplus [86]. We ran a two-level null model without any predictors in the hierarchical linear modelling software to examine whether there was a significant amount of variance in outcome across groups [87]. For customer satisfaction, the ratio of between-groups to total variance yielded an ICC (1) of 0.24, indicating that 24% of the total variance in customer satisfaction was due to group membership.

The result (see Table 4) showed that the moderating effect was significant (γ = −1.14, *p* < 0.01, 95% CI = [−1.840, −0.435]). Further, the pseudo-R2 showed that predictors explained 6% variance in customer satisfaction at the individual level and 3% at the group level. Thus, a mediating effect of life satisfaction on customer satisfaction via service-oriented OCB differed in conditions of low and high group competition climate, such that the high group competition climate alleviated the indirect effect. Thus, Hypothesis 4 was supported.

**Figure 2:**
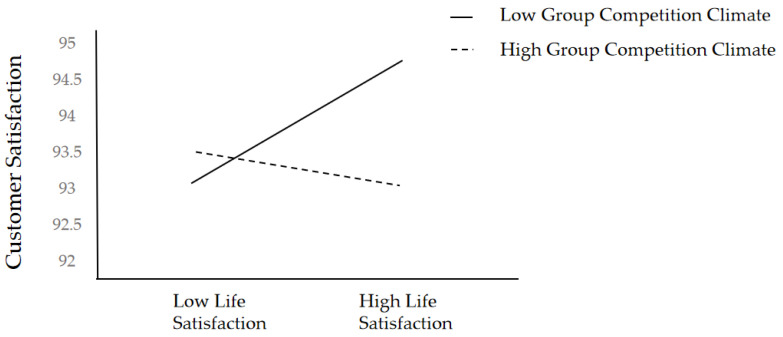
Group Competition Climate Moderating the Indirect Relationship Between Life Satisfaction and Customer Satisfaction.

We plotted this interaction effect (see Figure 2) within the moderated mediation model at conditional values of group competition climate (1 SD above and below the mean). Simple slope test result shows that the relationship between service-oriented OCB and customer satisfaction on the mediation path was significantly positive at lower levels of group competition climate (simple slope = 0.16, *t* = 2.05, *p* < 0.05, 95% CI = [0.008, 0.316]) but not at higher levels of group competition climate (simple slope = −0.04, *t* = −0.88, *p* > 0.05, 95% CI = [−0.149, 0.057]), which means that the correlation between service-oriented OCB and customer satisfaction was no longer significant in high competition groups.

## 5. Discussion

### 5.1. Summary of the Findings

Few enterprises fully appreciate how employees’ non-work experience will affect their service behavior towards customers. This study investigated the effect of life satisfaction on customer satisfaction and highlights its underlying mechanism and boundary conditions. The findings do not support a direct effect of life satisfaction on customer satisfaction but do support an indirect effect. In other words, employees’ overall life satisfaction has a significant indirect effect on customer satisfaction through service-oriented OCB. Furthermore, results show that the group competition climate buffers the indirect relationship between life satisfaction and customer satisfaction. When the group competition climate was low, the indirect effect of life satisfaction on customer satisfaction was significant, but when the team competition atmosphere was high, the indirect effect was no longer significant.

First, the results do not support our hypothesis that employees’ overall life satisfaction would positively affect customer satisfaction. There are two possible explanations for this result. On the one hand, the relationship between life satisfaction and customer satisfaction is far from being a simple model but instead has complex patterns, which may be affected by moderating variables such as personality or work attitude. It is likely that the effects of these variables on the relationship between life satisfaction and customer satisfaction offset each other, so the total effect is not significant.

On the other hand, the effect of life satisfaction on customer service needs to be transmitted through one or more mediator variables. Thus, the employee’s life satisfaction has no direct effect on customer satisfaction; rather, its entire effect is indirect.

Second, this study sought to identify an indirect effect of life satisfaction on customer satisfaction through service-oriented OCB, and this was supported by the results. The mediating role of service-oriented OCB on the life satisfaction–customer satisfaction link resonates with previous research which found that life satisfaction can predict organizational citizenship behavior [17] through job satisfaction [16,18] and then predict customer service quality [26,27,28].

Finally, our findings show that the group competition climate buffers the indirect relationship between life satisfaction and customer satisfaction. When the group competition climate was low, the indirect effect of life satisfaction on customer satisfaction was significant, but when the team competition atmosphere was high, the indirect effect was no longer significant. This finding complements previous research on the impact of competition on individual performance, which has produced inconsistent results, including positive effects [35], negative effects [30], and no significant correlation [29]. This study contributes to a growing body of evidence suggesting the detrimental effect of competition on individual performance.

### 5.2. Theoretical Contributions

This study makes three theoretical contributions. First, it expands understanding of the critical role played by employees’ non-work experience in organizational outcomes. According to the results of the previous meta-analysis, few studies have examined how employees’ life satisfaction relates to customer service [4]. This study fills the research gap by investigating the spillover effect of life satisfaction on customer satisfaction, thus contributing a new perspective from outside the workplace to the explanatory framework of customer service [4,34].

Second, this study enriches the understanding of the COR theory by showing the positive impact of life satisfaction on service-oriented OCB. Based on the work–family enrichment theory [13] and the COR theory [14], we hypothesize that individuals with higher life satisfaction can possess more work resources and positive affect [70] and have higher level of job satisfaction [71] and work engagement [19,20,21,22,23], which in turn will increase their extra work behaviors [24,25]. The results of our study support this hypothesis and broaden the understanding of the COR theory that it can be used to explain how personal resources impact service-oriented OCB.

Third, our study reveals a negative moderating effect of group competition climate on service performance, which contradicts the historical view of its positive effect [35]. In one study of Chinese hair salon staff, results suggested that the group competition climate improved the positive relationship between self-efficacy and service quality, with one possible explanation being that the subjects did not need to depend on each other at work [35]. The current study also employed subjects with high work independence; that is, the call center staff. However, our results demonstrated that the group competition climate impeded, rather than facilitated, the positive relationship between service-oriented OCB and customer satisfaction. Our findings complement previous investigations of the moderating effect of competition climate.

### 5.3. Practical Implications

This study also has significant practical implications. First, this paper provides enterprises with new perspectives for people-oriented management. According to our findings, an increase in employees’ life satisfaction is conducive to promoting service-oriented OCB, which shows that in addition to employees’ workplace experience, employees’ life experience may also affect critical organizational behaviors. Existing studies have discussed methods which enterprises might use to promote employees’ family life, including improving employees efficiency to reduce daily workload, creating a family-supportive organizational culture, and arranging emotion management training [88]. The current study suggests that organizations should expand their lens to include a broader array of non-work experiences, such as employees’ physical health [42], financial security [8], social relations, and leisure satisfaction [43].

Second, this study suggests that for enterprises with a high competition climate, the question of how to reduce the negative impact of competition on performance is a problem worthy of further exploration. A certain level of competition is beneficial to employee performance [89]. However, according to the findings of this study, a high-level group competition climate, does not strengthen but hinders the positive impact of life satisfaction on customer service quality. Therefore, we suggest that managers consider two factors to maintain a reasonable competitive level. One factor to be considered is the stress level of team members. According to the Yerkes–Dodson Law, there is an inverted U-shaped relationship between individual stress and performance [89]. Therefore, when an increase in pressure fails to positively affect performance, the peak of the competition curve appears, indicating that a further pressure level increase might be detrimental to performance. Another factor is the completion rate of individual or team goals. When the performance has been completed, or the team has outperformed the competitors, fewer incentive measures should be taken to promote competition, and more measures should be adopted to alleviate employees’ stress levels.

### 5.4. Limitations and Future Research

This study also has some limitations. First, the model used only provides a preliminary framework since the relationship between life satisfaction and customer satisfaction is too complicated. Past research has revealed that the mixed effects of the life-work link can be caused by personality traits. For example, a higher level of neuroticism was associated with negative spillover between family and work, while a higher level of extraversion was associated with a positive spillover effect [37]. Several studies also support the idea that individuals high in conscientiousness tend to control life–work cross-boundary interruptions in a way that aligns with their needs and identities [90,91]. Similarly, for those service employees high in conscientiousness, even if they are not happy in life, it is less likely that they will exhibit negative emotions in a service job, and thus the effect between life satisfaction and customer satisfaction is not directly significant. Future models can examine more mediating and moderating variables. For example, employees’ in-role behavior is a potential mediating variable because of its importance in job performance, and it should be tested as a mediating variable in future research. In addition, personality is a potential moderating variable. Previous research supports that trait affectivity and core self-evaluation can weaken the correlation between life satisfaction and job satisfaction [42].

Second, broader samples from various nations or cultures should be included in future research. Given that the context of our study was frontline service employees of an insurance company in China, it is necessary to apply our model in a Western cultural setting to increase the generalization of our results. Specifically, the variables in our model have cross-cultural differences as follows: (1) Life Satisfaction. Life satisfaction in individualistic cultures (e.g., Europe and America) is significantly higher than that in collectivist cultures (e.g., East Asian countries) [92]. (2) Life satisfaction–job satisfaction links may differ across cultures and individuals with different socioeconomic characteristics [11]. (3) Customer Experience. Special treatment benefits, reflecting social bonds, were more important for customers rated high on uncertainty avoidance and collectivism [93], indicating that employees need to do more work beyond job duties. In conclusion, future research can examine the life satisfaction–customer satisfaction link in a cross-culture context.

## 6. Conclusions

This study makes a preliminary exploration of the impact of overall life satisfaction on customer service quality. Building upon the work–family enrichment model and the COR theory, we predicted and found that life satisfaction has a positive effect on customer satisfaction indirectly through service-oriented OCB. Group competition climate buffered the positive relationships between life satisfaction and customer satisfaction, such that the links were not significant when the group competition climate was high. Our research explored the spillover relationship between the work and non-work domains from a top-down perspective, highlighting meaningful directions for future research on the impact of overall life experience on customer service performance within organizations.

## Figures and Tables

**Table 1:** Means, Standard Deviations, Pearson Correlations, and Scale Reliabilities.

Variable	*M*	*SD*	1	2	3	4	5	6
Employee/T1								
1. Age (in years)	26.21	3.16						
2. Gender			0.01					
3. Tenure (in months)	21.80	23.86	0.06	0.60 **				
4. Life satisfaction	4.20	1.19	0.01	0.07	0.06	(0.86)		
Employee/T2								
5. Service-oriented OCB	5.45	0.95	0.11	−0.03	−0.03	0.15 *	(0.96)	
6. Group competition climate	4.73	1.40	−0.07	−0.04	−0.06	0.04	0.51 **	(0.86)
Customer/T3								
7. Customer satisfaction	93.57	2.75	0.12 ^†^	−0.03	0.19 **	0.01	0.13 ^†^	0.08

Note. *N_individual_* = 209; *N_group_* = 29. Reliability estimates (coefficient alpha) are in parentheses on the diagonal. OCB = organizational citizenship behavior; Gender coded 1 = female, 0 = male; ^†^ *p* < 0.10, two-tailed. * *p* < 0.05, two-tailed. ** *p* < 0.01, two-tailed.

**Table 2:** Confirmatory Factor Analyses.

Model	*χ* ^2^	*df*	RMSEA	SRMR	CFI	TLI	Δ*χ*^2^ (Δ *df*)
Three-Factor Model	411.55	232	0.06	0.06	0.96	0.95	
Two-Factor Model	697.65	234	0.10	0.08	0.89	0.86	286.10 (2) **
Single-Factor Model	1199.94	235	0.14	0.12	0.78	0.72	502.29 (1) **

Note. *N* = 209. RMSEA = Root Mean Square Error of Approximation; SRMR = Standardized Root Mean Residual; CFI = Comparative Fit Index; TLI = Tucker–Lewis Index; ** *p* < 0.01.

**Table 3:** Individual Analyses Results for Testing Hypotheses 1 to 3.

Variables	Model 1		Model 2		Model 3	
	DV = Customer Satisfaction		Mediator = Service-Oriented OCB		DV = Customer Satisfaction	
	*B*	*SE*	*B*	*SE*	*B*	*SE*
Intercept	93.21	2.01	4.91 ***	0.70	91.09 ***	2.22
Control variables						
Gender	−0.38	0.54	0.31	0.19	−0.51	0.54
Age	0.02	0.08	−0.01	0.03	0.02	0.07
Tenure	0.02 *	0.01	−0.00	−0.00	0.02 *	0.01
Predictors						
Life satisfaction	−0.04	0.16	0.12 *	0.06	−0.09	0.16
Service-oriented OCB					0.43 *	0.20
*R* ^2^	0.04		0.04		0.06	

Note. *N* = 209. OCB = organizational citizenship behavior; * *p* < 0.05, two-tailed. *** *p* < 0.001, two-tailed. DV = dependent variable.

**Table 4:** Multilevel Analysis Results for Testing Hypothesis 4.

Variables	Model 4			
	Mediator = Service-Oriented OCB		DV = Customer Satisfaction	
	γ	*SE*	γ	*SE*
Intercept	0	0.06	93.95 ***	1.88
Control variables				
Gender			−0.39	0.50
Age			−0.02	0.07
Tenure			0.02 *	0.01
Predictors				
Life satisfaction	0.17 **	0.06	−0.04	0.17
Service-oriented OCB			0.35 ^†^	0.21
Group competition climate			−0.63 ^†^	0.38
Service-oriented OCB * Group competition climate			−1.14 **	0.43
σ^2^	0.79		7.51	
~*R*^2^ (individual level)			0.06	
~*R*^2^ (group level)			0.03	

Note. *N_individual_* = 209; *N_group_* = 29. γ = unstandardized coefficient obtained from hierarchical linear modelling. OCB = organizational citizenship behavior; ^†^ *p* < 0.10, two-tailed. * *p* < 0.05, two-tailed. ** *p* < 0.01, two-tailed. *** p < 0.001, two-tailed. DV = dependent variable.

## Data Availability

The employee self-reported data are available from the corresponding author upon reasonable request.
